# Evaluation of the difference-correction effect of the gamma camera systems used by easy Z-score Imaging System (eZIS) analysis

**DOI:** 10.1007/s12149-014-0807-z

**Published:** 2014-01-25

**Authors:** Yasushi Yamamoto, Masahisa Onoguchi, Kazunori Kawakami, Masuo Haramoto, Rei Wake, Jun Horiguchi, Hajime Kitagaki

**Affiliations:** 1 Department of Radiology, Shimane University Hospital, 89-1 Enya-cho, Izumo, Shimane 693-8501 Japan; 2 Department of Quantum Medical Technology, Graduate School of Medical Science, Kanazawa University, Kodatsuno 5-11-80, Kanazawa, Ishikawa 920-0942 Japan; 3 Department of Psychiatry, Shimane University School of Medicine, 89-1 Enya-cho, Izumo, Shimane 693-8501 Japan; 4 Department of Radiology, Shimane University Faculty of Medicine, 89-1 Enya-cho, Izumo, Shimane Japan

**Keywords:** ^99m^Tc-ECD, ^99m^Tc-HMPAO, SPM, eZIS, Image correction

## Abstract

**Objective:**

We examined the difference of the effect by data to revise a gamma camera difference. The difference-correction method of the camera is incorporated in eZIS analysis.

**Methods:**

We acquired single photon emission computed tomography (SPECT) data from the three-dimensional (3D) Hoffman brain phantom (Hoffman), the three-dimensional brain phantom (3D-Brain), Pool phantom (pool) and from normal subjects (Normal-SPECT) to investigate compensating for a difference in gamma camera systems. We compared SPECT counts of standard camera with the SPECT counts that revised the difference of the gamma camera system (camera). Furthermore, we compared the “Z-score map (Z-score)”. To verify the effect of the compensation, we examined digitally simulated data designed to represent a patient with Alzheimer’s dementia. We carried out both eZIS analysis and “Specific Volume of interest Analysis (SVA)”.

**Results:**

There was no great difference between the correction effect using Hoffman phantom data and that using 3D-Brain phantom data. Furthermore, a good compensation effect was obtained only over a limited area. The compensation based on the pool was found to be less satisfactory than any of the other compensations according to all results of the measurements examined in the study. The compensation based on the Normal-SPECT data resulted in a Z-score map (Z-score) for the result that approximated that from the standard camera. Therefore, we concluded that the effect of the compensation based on Normal-SPECT data was the best of the four methods tested.

**Conclusions:**

Based on eZIS analysis, the compensation using the pool data was inferior to the compensations using the other methods tested. Based on the results of the SAV analysis, the effect of the compensation using the Hoffman data was better than the effect of the compensation using the 3D-Brain data. By all end-point measures, the compensation based on the Normal-SPECT data was more accurate than the compensation based on any of the other three phantoms.

## Introduction

It has been reported that in the interpretation of cerebral blood flow images from either positron emission tomography (PET) or single photon emission computed tomography (SPECT), a diagnostic improvement is realized by statistical image analysis using statistical parametric mapping (SPM) [1, 2] or three-dimensional stereotactic surface projections (3D-SSP) [3–5]. For the statistical image analysis, a normal database (NDB) constructed from the images of a group of normal subjects is necessary. It is used to make a reference image to which the image of a patient is compared [6–8]. Using a NDB constructed from one gamma camera system (camera) to analyze a patient scan acquired on a different camera would appear to risk error because of differences in the properties of the detectors and in the structure of the collimators used for different cameras.

Using 3D-SSP, after a subject’s brain image was aligned, spatially normalized into a standard space, and smoothed, we measured counts from each pixel of the cerebral surface for 6 pixels in the vertical direction of the cortex. We then extracted the maximum count to the corresponding cerebral surface and performed imaging [9]. Therefore, the difference in the cameras and anatomical error are thought to be solved problems. Consequently, compensation for the difference in the cameras (the camera which created the NDB and the camera which carried out the clinical study) is not necessary [9].

In contrast, in the SPM analysis, the high-frequency component is removed by a smoothing filter [10, 11], and we were unable to employ the NDB if we did not compensate for the difference in the cameras. Therefore, Matsuda et al. [5, 11–14] developed user interface software that incorporated the difference-correction method into the SPM2 analysis algorithm. It is the analytical method called easy Z-score imaging system (eZIS analysis).

Matsuda et al. [15, 16] also developed Alzheimer’s dementia diagnosis support software called specific volume of interest analysis (SVA). SVA uses voxel of interest (VOI) of the disease specific region as a mask (SVA mask), and three diagnosis support indices are calculated by regional Z-score. These indices are usually calculated after a compensation for the difference in the cameras.

The difference compensation within the eZIS analysis functions as follows: first, we collect Hoffman Brain phantom (Hoffman) data with the camera that created the NDB and the camera used for the clinical study. Second, we multiply “Count Ratio” in “Subjects data”, and correct it so that it is as if the data were collected with the gamma camera that created the NDB [11]. “Count Ratio” is the ratio of the Hoffman data that we collected with the camera we used for the clinical studies and the Hoffman data that we collected with the camera which created the NDB. Therefore, the eventual results of an analysis are thought to depend on the compensation effect (correction effect of the camera difference). In an eZIS analysis, the Hoffman phantom is usually used to generate difference-correction data. However, there have not been reports on the compensation effect when other phantoms are used.

## Objectives

After having confirmed that a SPECT image varied according to a collector, we examined the difference of the correction effect by the data to revise a camera difference, in eZIS analysis. Also, we created data for difference corrections of the camera from the data of normal subjects, and examined them.

This study received the approval of the ethics committee of Shimane University School of Medicine.

## Materials and methods

### Construction of the normal subjects’ SPECT image data

#### The background of the normal subjects

We recruited normal volunteers who did not suffer from brain disease and performed the cerebral blood flow SPECT examination using ^99m^Tc-ethyl cysteinate dimer (^99m^Tc-ECD) on 20 subjects from March 2011 to September 2011. We recruited a healthy volunteer to make a NDB. And we used it for making data to revise the difference in the camera. The normal-subject group consisted of 10 persons in their 60 s (age 65.7 ± 2.2, 5 men, 5 women), and 10 in their 70 s (age 74.1 ± 2.2, 5 men, 5 women). The Hasegawa-style simple intelligence study (HDS-R) mean score was 28.5 ± 1.1 for those in their 60 s, and was 28.4 ± 1.6 for those in their 70 s. The mini-mental state examination (MMSE) mean score was 29.2 ± 1.7 for those in their 60 s, and was 28.9 ± 1.7 for those in their 70 s. It was necessary for us to know a subject’s mean cerebral blood flow (mCBF, ml/100 g/min) to verify the normality of the brain–blood flow of the persons to be included in the NDB. We determined the mean cerebral blood flow by the Patlak Plot method which is a noninvasive cerebral blood flow measurement using non-blood sampling [17]. The mCBF was 43.4 ± 4.2 for those in their 60 s, and 41.8 ± 4.4 for those in their 70 s.

We also recruited 32 other volunteers who showed normal findings in a previous brain health examination that had been conducted to check for signs of cerebral infarctions and other problems. These 32 volunteers were needed to allow one of the different compensation methods to be used and also for a NDB, as in the case with ^99m^Tc-ECD. We acquired cerebral blood flow SPECT images using ^99m^Tc–hexamethylpropylene amine oxime (^99m^Tc-HMPAO) of these subjects from March 2010 to December 2010. The normal-subject group was comprised of 10 persons in their 50 s (mean age 55.2 ± 3.0, 4 men, 6 women), 12 in their 60 s (mean age 63.8 ± 2.9, 7 men, 5 women), and 10 in their 70 s (mean age 73.2 ± 2.4, 5 men, 5 women). Okabe’s Scale totals were 48.9 ± 3.0 for those in their 50 s, 47.0 ± 5.7 for those in their 60 s, and 45.4 ± 6.9 for those in their 70 s. The Okabe test is a modified and simplified version of the Wechsler memory scale and consists of 4 subscales: information, mental control, digit span, and associative learning. The full scores on these 4 subscales amount to a total 60 points. The classification criteria are <10 points, severe dementia; 10–19 points, moderate dementia; 20–29 points, mild dementia (or notable mental aging) [18].

We again determined the mCBF by the Patlak plot method; the mCBF was 47.3 ± 5.0 for those in their 50 s, 43.4 ± 10.4 for those in their 60 s, and 41.7 ± 4.4 for those in their 70 s.

In these two cases, there were no cases with abnormalities such as asymptomatic cerebral infarction in MRI examination (MRI: signa HDX 3.0T and signa 1.5T, General Electric), and the degree of atrophy was not excessive for their age.

#### A SPECT image collection condition and the reconstruction parameters for normal subjects

We performed the cerebral blood flow SPECT examination using 740 MBq of ^99m^Tc-ECD. The cameras used for imaging were a PRISM IRIX/Odyssey FX camera/image processor (Philips Healthcare, Amsterdam, Netherlands) and an E.CAM/e.soft camera/image processor (Siemens Healthcare, Munich, Germany). However, the tracer was administered only once. First, we collected SPECT data using the IRIX. Less than 10 min elapsed after collection of the data with the IRIX before we started to collect data with the E.CAM. The acquisition parameters with the IRIX camera were energy window 140 keV ± 7.5 %, and data sampling angle 5° step with 72 views (35 s/view). A Butterworth filter was used as a filtered back projection method for SPECT image reconstruction at 0.75 cycle/cm. Attenuation compensation was performed using Chang’s method with 0.1 cm^−1^. Those of E.CAM camera: 140 keV ± 10 %, 4° step/90 views (15 s/view), Butterworth filter 0.45 Nyquist frequency, filtered back projection, attenuation compensation by the Chang’s method with 0.1 cm^−1^. The cut-off frequency of the respective Butterworth filters had been set so that the resolution of the image collected by one device was equal to the resolution of the image collected by the other cameras [19]. Both cameras were equipped with low-energy high-resolution collimators.

We performed another cerebral blood flow SPECT examination using 740 MBq of ^99m^Tc-HMPAO. The SPECT image collection condition and reconstruction parameters were the same as those for ^99m^Tc-ECD.

### Acquisition and processing of phantom data to resolve the camera differences (Fig. 1)

We used the Hoffman, the three-dimensional brain phantom (3D-Brain) and the Pool phantom (pool) data to construct the respective difference-compensation data. The Hoffman differs from the human cranium and its brain because the phantom is cylindrical and does not have an *ossa cranii*. To resolve these differences, Iida et al. developed a phantom called the 3D-Brain. A bone equivalent fluid (K_2_HPO_4_) is enclosed in the *ossa cranii* of the 3D-Brain. The cerebral parenchyma is constructed from computed tomography (CT) images of normal subjects. A radioactive tracer can be enclosed in the *substantia grisea* [20]. As for the pool, brain parenchyma of Hoffman is excluded; therefore the pool is a simpler phantom than the Hoffman. We put 0.3 MBq/ml of ^99m^TcO4^−^ in each phantom. We then acquired SPECT images with both the IRIX camera and the E.CAM camera three times for each phantom. The SPECT imaging conditions and image reconstruction conditions were the same as those for the normal subjects. We performed anatomical standardization using a template suitable for each phantom. The data for compensation were constructed from the average of the three data sets.

**Fig. 1:**
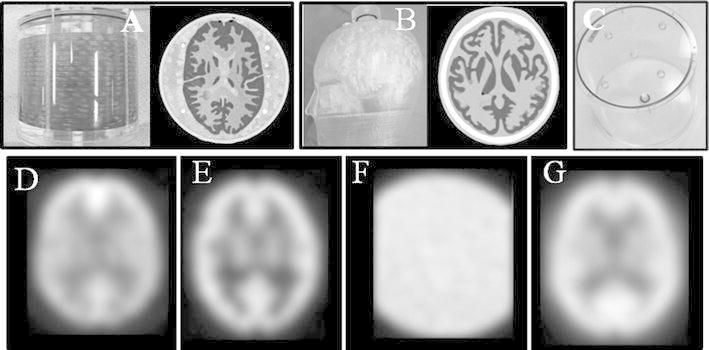
Photographs of three phantoms and their images. A Hoffman phantom and its CT image (**a**). A 3D-Brain phantom and its CT image (**b**). A pool phantom (**c**). All of four images below show the SPECT images for compensations that were obtained by the IRIX camera. The Hoffman data (**d**), 3D-Brain data (**e**), pool data (**f**), and Normal-SPECT data (**g**)

In addition to using each of the three phantoms, we also used live subjects. That is, we acquired images to resolve the differences between the two cameras from the average of a set of normal-subject brain–blood flow SPECT images. Furthermore, from normal subjects’ average SPECT image data, we prepared data (Normal-SPECT data) for difference compensation of the cameras. The procedure was to first realign the image, and then anatomically standardize it using a template for ECD. And we prepared Normal-SPECT data for both cameras (IRIX and E.CAM).

In the case of ^99m^Tc-HMPAO, we prepared compensation data (Normal-SPECT data) using 22 subjects’ (12 in their 60 s and 10 in their 70 s) average SPECT image data similar to the case for ^99m^Tc-ECD.

### Difference in SPECT images of the gamma cameras

The data reconstituted from the normal subjects’ (*n* = 20) data that used ^99m^Tc-ECD obtained with an E.CAM camera were used as the reference data (referred to below as “E.CAM Ref ECD data”). Similarly, those for the IRIX camera are referred to as “IRIX data”. The procedure for producing what we called “E.CAM Ref ECD data” is different from that for the above-mentioned “Normal-SPECT data”. We collected the raw data of normal subjects using the E.CAM and carried out attenuation correction and reconstruction. We applied an anatomical standardization and smoothing. The result was called the “E.CAM Ref ECD data” (*n* = 20). We used eZIS analysis (SPM2) for the anatomical standardization and smoothing. We also collected normal-subject raw data with the IRIX and prepared IRIX data (*n* = 20) in the same manner.

We performed a two-sample *t* test (height threshold *P* value 0.05, uncompensated, *P* value adjustments to none, extent threshold 50 voxels) between the E.CAM Ref ECD data and IRIX data by the statistical mode of SPM8. The SPM8 software is the current version of SPM. We thus assessed the difference in SPECT images (the normalized SPECT counts) acquired with the two cameras. Then, in all two-sample *t* tests, we used the statistical mode of SPM8 and used the same procedure described above.

### Construction of data that artificially emphasize the compensation effect

We reconstructed the raw data from the normal subjects acquired using the IRIX without attenuation compensation (referred to below as IRIX AC^−^ data) to clearly examine the effect of the compensation, that is, so that the relative difference in the counts of the SPECT image data from the two cameras would be increased. We revised the difference of the cameras for IRIX AC^−^ data. At all voxels, we calculated the count ratio of “Data (with attenuation compensation) to use for the difference compensation of the cameras which we collected in E.CAM” and “Data (without attenuation compensation) to use for the difference compensation of the cameras which we collected in IRIX”. We multiplied every voxel of “IRIX AC^−^ data” in the count ratio. We compared the E.CAM Ref ECD data with the IRIX AC^−^ data that revised the difference of the cameras.

### Evaluation of the effect of the compensation by the statistical significance test result images

We constructed statistical significance test result images (referred to below as “Decrease” and “Increase”) by performing a two-sample *t* test between the compensated IRIX AC^−^ data and E.CAM Ref ECD data. For the IRIX AC^−^ data, we used each of the following compensation methods: “Hoffman data, 3D-Brain data, pool data, and Normal-SPECT data,” and examined the effect from “Decrease image” and “Increase image” as reference E.CAM Ref ECD data. The “Decrease image” shows those pixels where counts decreased in a statistically significant way. The “Increase image” shows those pixels where counts increased in a statistically significant way. We then visually examined the effect of the compensation. Also, we compared the IRIX AC^−^ data with E.CAM Ref ECD data.

### Evaluation of the compensation effect by the Z-score

We created a NDB (E.CAN NDB) using the normal subject raw data that we collected using the E.CAM. We acquired a Z-score (map), which we define as a standard Z-score map, by performing the eZIS analysis using the E.CAM NDB with the E.CAM Ref ECD data. And we acquired a Z-score map by eZIS analysis using E.CAM NDB with the compensated IRIX AC^−^ data. Also, we acquired the Z-score (map) by an eZIS analysis using E.CAM NDB with IRIX AC^−^ data to not compensate for the difference in the cameras. If the difference compensation is appropriate, the Z-score should be similar to that of the standard Z-score map. We calculated the average Z-score of the “Decrease and Increase” for the central area (Fig. 3a) and for the marginal area (Fig. 3b) and evaluated the correction effect. We performed a multiple comparison by the Tukey–Kramer method to test the statistical significance. A central area and the marginal area divide a significant difference image, which we made by two-sample *t* test between E.CAM Ref ECD data and IRIX AC^−^ data into two regions. The division is made using software for multipurpose image analyses named DRIP.

### Evaluation of the correction by the correlation coefficient of the SPECT count

We compensated for the IRIX data by multiplying the original IRIX data by the image of the ratio of counts of difference-compensation data collected by E.CAM and constructed with attenuation compensation (Hoffman data, 3D-Brain data, pool data, Normal-SPECT data) and counts of difference-compensation data collected by IRIX and constructed with attenuation compensation. We calculated the correlation coefficient of the SPECT count of “E.CAM Ref ECD data” and that of “Adjusted IRIX data”, and thereby evaluated the correction effect. That is we examined how similar the adjusted IRIX data were to the E.CAM Ref ECD data.

Also, when we did not revise the IRIX data, we examined them. The correlation coefficient was calculated between every region of the same subjects. We set the regions using Level 2 of “voxel-based Analysis Stereotactic Extraction Estimation (vbSEE)” [21] developed by Mizumura; the six regions targeted for evaluation were: all lobes, frontal lobe, occipital lobe, temporal lobe, anterior lobe, sub-lobar. (We calculated right lobe and left lobe at the same time). We used Matlab R2009b (MathWorks, Natick, American MA state) for the calculation of the correlation coefficient. We used a two-way factorial analysis of variance to test the statistical significance of the coefficient of correlation.

### Construction of Alzheimer’s dementia digital simulation data

We constructed a SPECT image simulating Alzheimer’s dementia for both cameras (the IRIX AD and the E.CAM AD) from the SPECT images of a single subject (female; 63 years old; HDS-R, 30; MMSE, 30). IRIX AD data and E.CAM AD decreased 20 % of the SPECT counts of the SVA mask coordinate, and made it. This procedure was performed using a free software Daemon research image processor. (DRIP, multipurpose image analysis software; Fujifilm RI Pharma Ltd in Tokyo; Japan).

### Compensation effect evaluation by Z-score map where eZIS analyzed Alzheimer’s dementia digital simulation data

We corrected the IRIX AD data, and performed eZIS analysis using E.CAM NDB, and obtained a Z-score map. Also, we made IRIX AD data without an attenuation compensation, and acquired a Z-score map by eZIS analysis using the E.CAM NDB. We acquired a Z-score map by the eZIS analysis using IRIX NDB on IRIX AD data. We acquired a Z-score map by the eZIS analysis using E.CAM NDB on E.CAM AD data. We then visually examined these Z-score maps. We conducted SVA analysis, and obtained severity (Se), extent (Ex) and ratio. We compared each value, and evaluated a correction effect. We can determine the “Extent” and “Severity” of the cerebral blood flow decrease by an SVA analysis. We can also determine the ratio of the brain–blood flow decreased region over the whole brain by SVA analysis.

### Examination when healthy subject data group is different from a group with Normal-SPECT data

The effect of the compensation using Normal-SPECT data was assessed using the SPECT image data group of ^99m^Tc-HMPAO as the data group different from the Normal-SPECT data constructing group. We performed a two-sample *t* test between IRIX data using ^99m^Tc-HMPAO and the E.CAM Ref data using ^99m^Tc-HMPAO (referred to below as E.CAM Ref PAO data), between IRIX AC^−^ data using ^99m^Tc-HMPAO and E.CAM Ref PAO data, and between “IRIX AC^−^ data using ^99m^Tc-HMPAO that were compensated for using Normal-SPECT data” and E.CAM Ref PAO data. We then constructed the statistical significance test result images and visually assessed the effect of the compensation for difference in the cameras. E.CAM Ref PAO data, IRIX data of ^99m^Tc-HMPAO and IRIX AC^−^ data of ^99m^Tc-HMPAO were constructed from the 10 normal subjects in their 50 s belonging to the group that was not used for constructing Normal-SPECT data of ^99m^Tc-HMPAO for the compensation of the difference in cameras.

## Results

### Difference in SPECT images of the gamma cameras

**Fig. 2:**
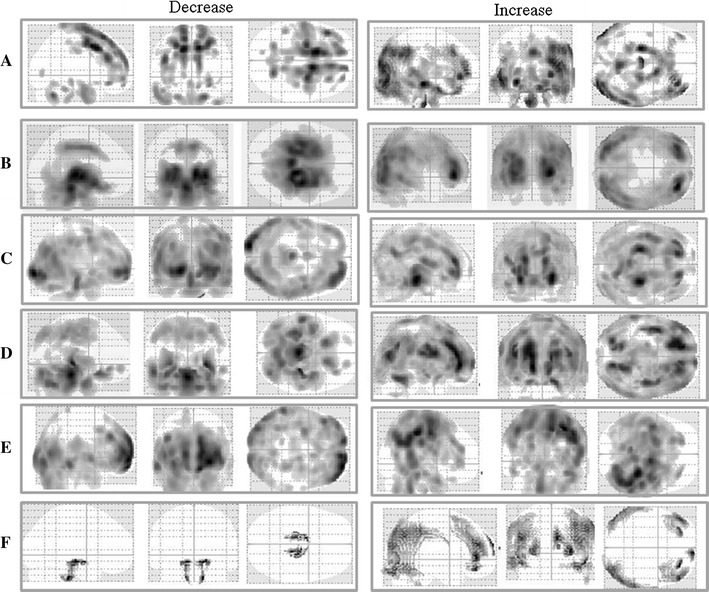
Maximum intensity projection (MIP) of SPM8 result for each two-sample *t* test on the basis of E.CAM Ref ECD data by ^99m^Tc-ECD SPECT. MIPs show the increase and decrease for IRIX data (**a**), IRIX AC^−^ data (**b**), compensated IRIX AC^−^ data by Hoffman (**c**), compensated IRIX AC^−^ data by 3D-Brain (**d**), compensated IRIX AC^−^ data by pool (**e**) and compensated IRIX AC^−^ data by Normal-SPECT (**f**), respectively

Figure 2a, show the results of the two-sample *t* test between the E.CAM Ref ECD data and IRIX data of the normal-subject group. “Decrease” means the region in which the counts of the IRIX data are lower than those of the E.CAM Ref data. “Increase” means the region in which the counts of the IRIX data are higher than those of the E.CAM Ref data. The counts of the IRIX data were lower than those of the E.CAM Ref ECD data in the frontal lobe. Conversely, the counts of the IRIX data were higher than those of the E.CAM Ref ECD data in the parietal lobe, temporal lobe and occipital lobe.

### Evaluation of the effect of the compensation by the statistical significance test result images

Figure 2b, show the result of the two-sample *t* test between the E.CAM Ref ECD data and IRIX AC^−^ data. In the central area, the counts of the IRIX AC^−^ data were significantly lower than those of the E.CAM Ref ECD data. Conversely, in the marginal area, the counts of the IRIX AC^−^ data were significantly higher than those of the E.CAM Ref ECD data. In Fig. 2c–f, we show the results of the two-sample *t* test between the E.CAM Ref ECD data and IRIX AC^−^ data that were compensated for by the respective difference-compensation data. “Increase image” is an image of the area that determined that “IRIX AC^−^ data which compensated for the difference in the cameras” are bigger than “E.CAM Ref ECD data”. “Decrease image” is an image of the area that determined that “IRIX AC^−^ data which compensated for the difference of the cameras” are smaller than “E.CAM Ref ECD data”. In the “Decrease image” of the central area, the difference in the count became less and the number of visualized regions was decreased as compared with the case without the compensation (Fig. 2b). In this way, we confirmed the effect of the compensation for the in difference of cameras. According to the “Increase image” (Fig. 2d), which was compensated for using 3D-Brain data, we confirmed that in the frontal lobe there were regions in which the difference in the count increased as compared with the case without compensation.

From the visual assessment of the statistical significance test result images, it can be seen that the difference in count was the smallest in the compensation using Normal-SPECT data (Fig. 2f).

### Evaluation of the compensation effect by the Z-score

**Fig. 3:**
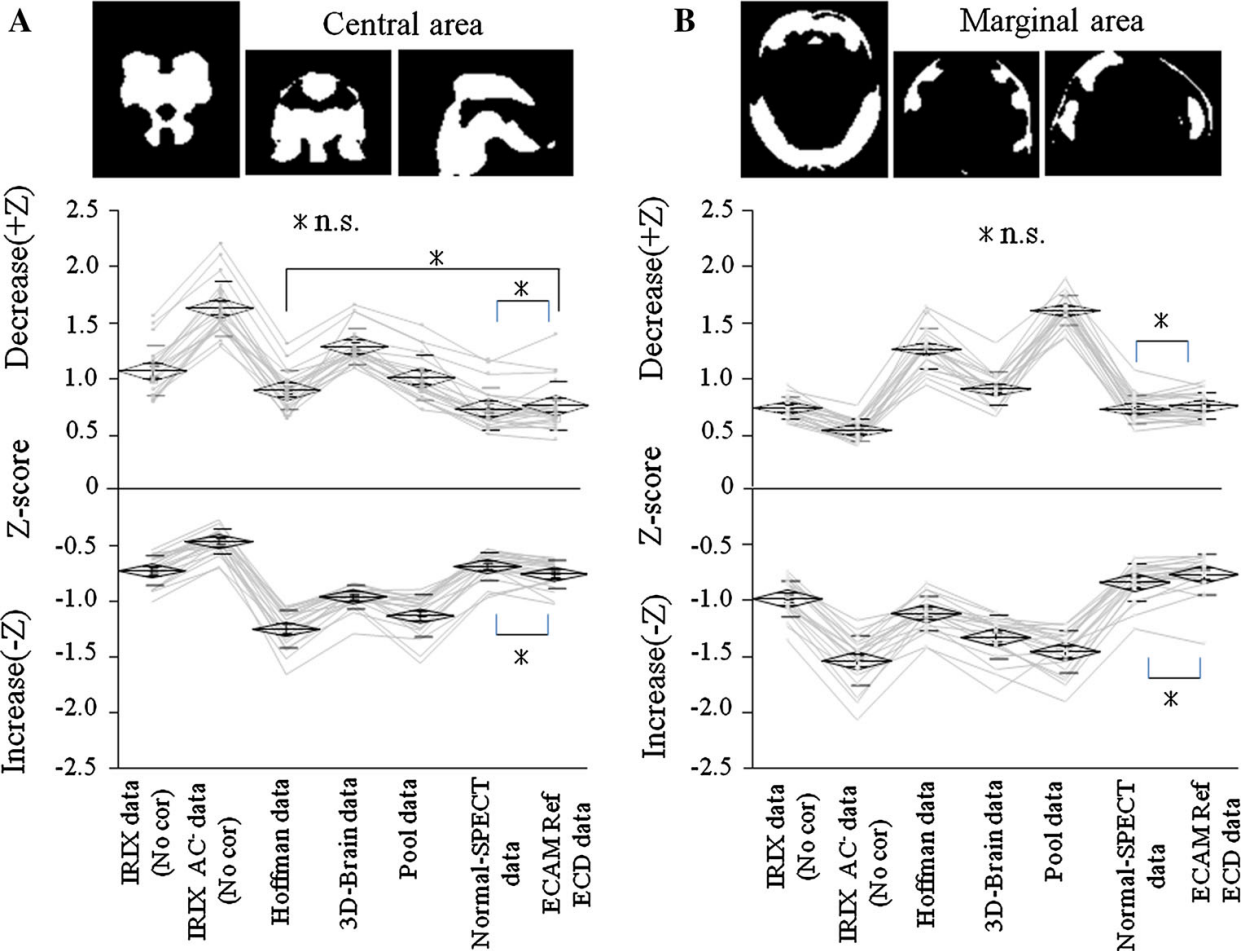
The binarized images of Fig. 2b show the central (**a**) and marginal (**b**). The Z-score below shows both the increase and decrease that were analyzed by each of the compensation methods. Each *polygonal line* of the Z-score corresponds to the same subjects. *Asterisk* n.s. (*P* ≥ 0.05)

Figure 3 displays the Z-score of the central area of the decrease and increase images, and that of the marginal area decrease and increase images. (The Z-score is on the vertical axis). A horizontal axis, “IRIX data (No cor)” was the case of the IRIX data that do not correct the difference in the camera. “IRIX AC^−^ data (No cor)” was the case of the IRIX AC^−^ data that do not correct the difference of the camera. “E.CAM Ref ECD data” was the case of the E.CAM Ref ECD data. “Hoffman data”, “3D-brain data”, “pool data” and “the normal-SPECT data” were the cases of the adjusted IRIX AC^−^ data using each type of correction data. The NDB used in all analyses is “E.CAM NDB”.

Table 1 shows the average and standard deviation (mean ± SD) of Z-score. In the decrease side of the central area, Normal-SPECT data (0.7440 ± 0.190) and Hoffman data (0.908 ± 0.168) did not have ECAM Ref ECD data (0.774 ± 0.218) and showed a significant difference. In the “Decrease” of the central area, the most ineffective was the compensation using 3D-Brain data (1.298 ± 0.160). Only Normal-SPECT data (−0.683 ± 0.129) did not have E.CAM Ref ECD data (−0.752 ± 0.122) and the significant difference that was usual in the increase side. Hoffman data (−1.245 ± 0.167) had the least correction effect. In the marginal area, only Normal-SPECT data (0.741 ± 0.125, −0.833 ± 0.160) did not have a normal value and a significant difference with either the decrease side or increase side. In pool data (1.617 ± 0.133, 1.450 ± 0.191), the decrease side of the marginal area and the increase side of the marginal area had no compensation effect.

**Table 1:** The average and standard deviation (mean ± SD) of the Z-scores in central area (A) and marginal area (B) analyzed by each of the compensation methods

Correction	A, central area		B, marginal area	
	Decrease	Increase	Decrease	Increase
IRIX data (No cor)	1.076 ± 0.221	−0.772 ± 0.135	0.752 ± 0.093	−0.979 ± 0.166
IRIX AC^−^data (No cor)	1.637 ± 0.245	−0.461 ± 0.113	0.553 ± 0.095	−1.530 ± 0.223
Hoffman data	0.908 ± 0.168	−1.245 ± 0.167	1.275 ± 0.179	−1.108 ± 0.153
3D-Brain data	1.298 ± 0.160	−0.953 ± 0.104	0.918 ± 0.146	−1.332 ± 0.191
Pool data	1.019 ± 0.204	−1.126 ± 0.186	1.617 ± 0.133	−1.450 ± 0.191
Normal-SPECT data	0.744 ± 0.190	−0.683 ± 0.129	0.741 ± 0.125	−0.833 ± 0.160
ECAM Ref data	0.774 ± 0.218	−0.752 ± 0.122	0.770 ± 0.118	−0.761 ± 0.177

Mean ± SD

### Evaluation of the correction by the correlation coefficient of the SPECT count

**Fig. 4:**
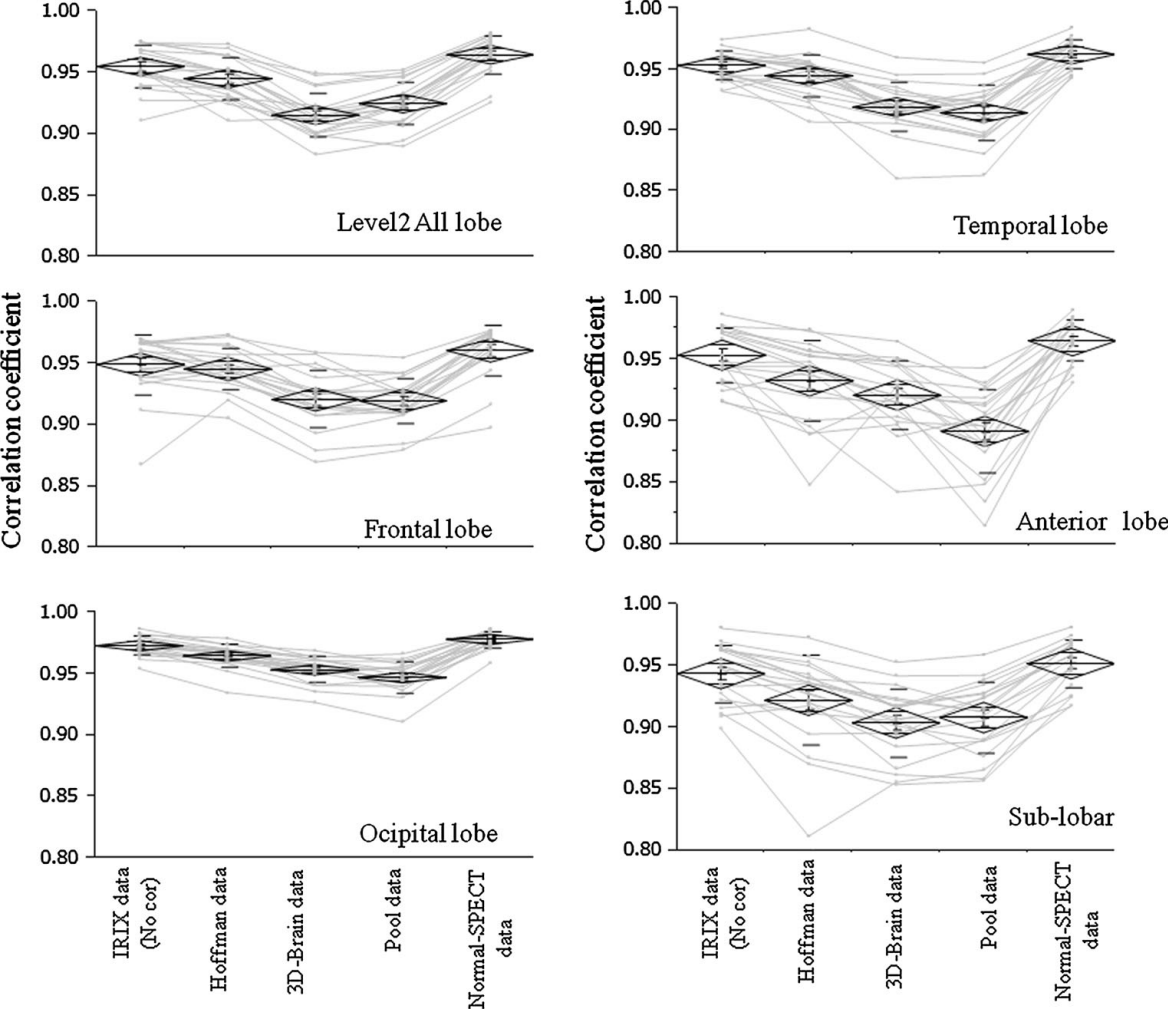
The correlation coefficient of the Z-scores by each of the compensation methods using vbSEE (Level 2) that simultaneously analyzed both right lobe and left lobe. Each *polygonal line* of each of the compensation methods corresponds to the same subject

“IRIX data (No cor)” on the horizontal axis in Fig. 4 were cases without compensation for the difference in cameras. “Hoffman data”, “3D-Brain data”, “Pool data”, “Normal-SPECT data” were cases of corrected IRIX data that used each of the respective correction data. The vertical axis shows the mean correlation coefficient (correlation coefficient of each of the IRIX data SPECT counts and E.CAM Ref ECD data SPECT counts).

We show the mean and standard deviation (mean ± SD) of the correlation coefficients in Table 2. In all regions, when we used Normal-SPECT data, the correlation coefficient was big (0.963 ± 0.016). The correlation coefficient became smaller in the order of IRIX data (0.954 ± 0.019), Hoffman data (0.942 ± 0.025), 3D-Brain data (0.922 ± 0.022), and pool data (0.917 ± 0.023). We compared the correlation coefficient of every region. In occipital lobe (0.963 ± 0.010) and posterior lobe (0.965 ± 0.011), the coefficient of correlation was bigger than in other regions, and the standard deviation was small. In anterior lobe (0.932 ± 0.027), and sub-lobar (0.926 ± 0.028), the coefficients of correlation were smaller than for other regions, and the standard deviations were big. There was a statistically significant difference between all difference-correction data. Furthermore, within a type of data set there was a statistical significance between the value differences for all pairs of regions.

**Table 2:** The average and standard deviation (mean ± SD) of the correlation coefficients between Z-scores and each of the compensation methods on the basis of E.CAM Ref ECD data

Correction	Level 2 all	Frontal	Occipital	Temporal	Anterior	Sub-lobar	Mean
IRIX data (No cor)	0.955 ± 0.017	0.949 ± 0.025	0.973 ± 0.008	0.953 ± 0.012	0.953 ± 0.021	0.944 ± 0.023	0.954 ± 0.019
Hoffman data	0.945 ± 0.020	0.946 ± 0.017	0.965 ± 0.009	0.945 ± 0.017	0.932 ± 0.033	0.922 ± 0.036	0.942 ± 0.025
3D-Brain data	0.915 ± 0.018	0.921 ± 0.023	0.953 ± 0.010	0.919 ± 0.020	0.921 ± 0.028	0.903 ± 0.028	0.922 ± 0.022
Pool data	0.925 ± 0.017	0.920 ± 0.018	0.947 ± 0.012	0.914 ± 0.022	0.891 ± 0.034	0.908 ± 0.029	0.917 ± 0.023
Normal-SPECT data	0.964 ± 0.015	0.960 ± 0.020	0.978 ± 0.006	0.962 ± 0.012	0.965 ± 0.017	0.952 ± 0.019	0.963 ± 0.016
Mean	0.941 ± 0.017	0.939 ± 0.021	0.963 ± 0.010	0.939 ± 0.017	0.932 ± 0.027	0.926 ± 0.028	

All areas were automatically divided by vbSEE

Mean ± SD

### Compensation effect evaluation by Z-score map where eZIS analyzed Alzheimer’s dementia digital simulation data

**Fig. 5:**
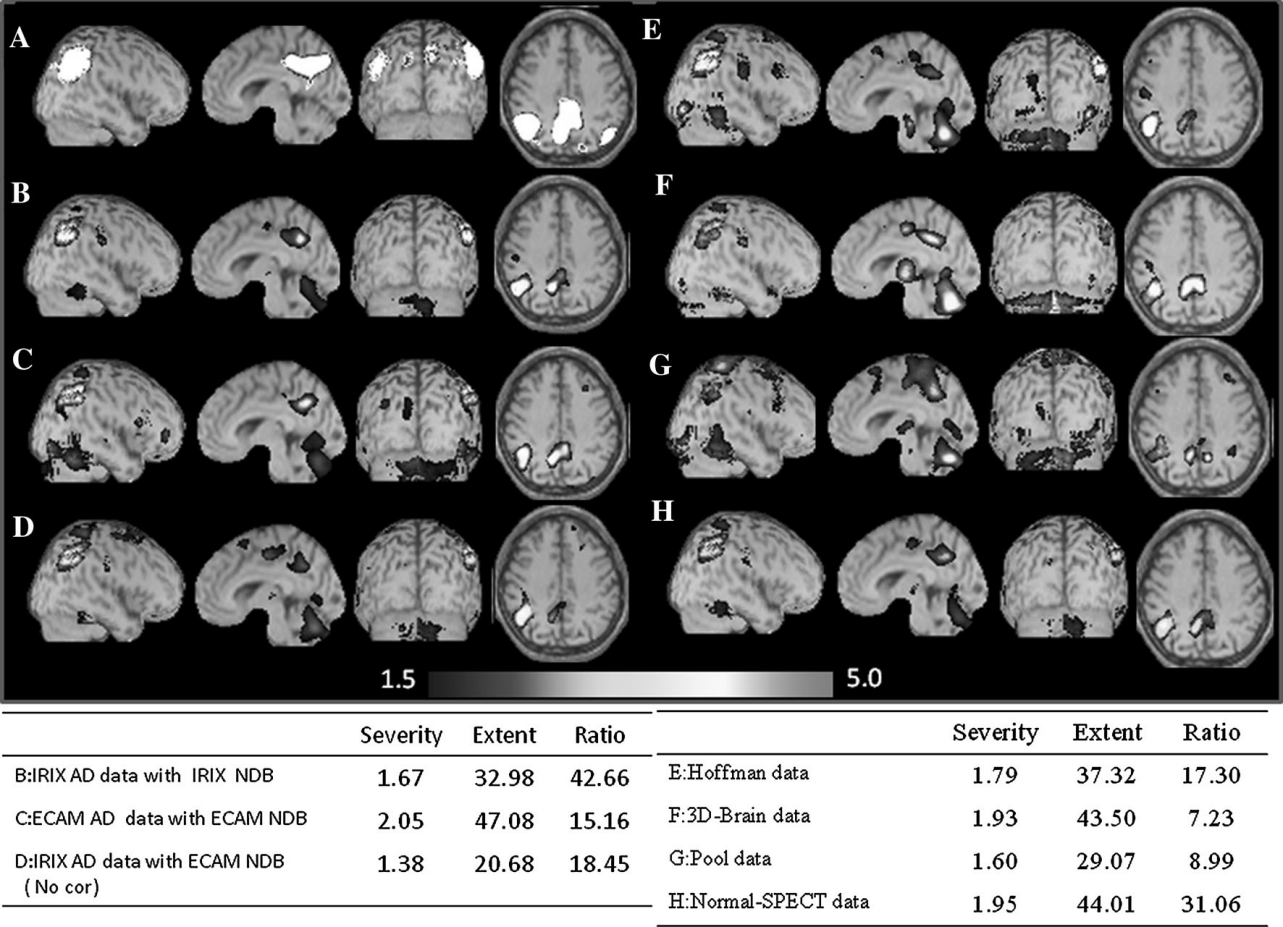
The above images show the Z-score map calculated by the eZIS analysis. The simulation image (**a**) intentionally decreased the SPECT counts using SVA mask processing (as shown in *white*) for the disease specific regions to simulate the AD. Others are the Z-score map images for IRIX AD data using IRIX NDB (**b**), E.CAM AD data using E.CAM NDB (**c**), IRIX AD data using E.CAM NDB (**d**), compensated IRIX AD data by Hoffman data using E.CAM NDB (**e**), compensated IRIX AD data by 3D-Brain data using E.CAM NDB (**f**), compensated IRIX AD data by pool data using E.CAM NDB (**g**), and compensated IRIX AD data by Normal-SPECT data using E.CAM NDB (**h**). The table below shows the index values (Severity, Extent and Ratio) analyzed by SVA from the Z-score map

In Fig. 5, we show the Z-score map of the IRIX AD data and that of E.CAM AD data and the results of the SVA analysis. To reduce noise, the minimum threshold value was set to 1.5. Figure 5a shows the disease specific regions that decreased the SPECT counts (SVA mask) in white. Figure 5b shows the Z-score map that analyzed the IRIX AD data in combination with the IRIX NDB. Figure 5c shows the Z-score map that analyzed E.CAM AD data in combination with the E.CAM NDB (standard Z-score map). The Z-score map varied according to the type of camera. Figure 5d shows the Z-score map that analyzed the IRIX AD data but does not revise the difference in the cameras in combination with the E.CAM NDB. The difference in the cameras compensates for the IRIX AD data using Fig. 5e Hoffman data, Fig. 5f 3D-Brain data, Fig. 5g pool data, and Fig. 5h Normal-SPECT data and shows the Z-score map which we analyzed in combination with the E.CAM NDB. SVA analyzes each Z-score map and shows the obtained Se, Ex, Ratio in Fig. 5, lower section. The difference in the cameras compensates for the IRIX AD data in “Hoffman data”, “3D-Brain data”, and “Pool data”, and the Z-score map that analyzed eZIS analysis had a bigger Z-score of the cerebellum region than the standard Z-score map, and the Z-score of the posterior part of the cingulate gyrus was different. The Z-score map that was the nearest to the standard Z-score map was the Z-score map where the gap between the camera revised the IRIX AD data in Normal-SPECT data (Fig. 5h). Without the compensation for the difference in the cameras (Fig. 5d), the Z-score of the cingulate gyrus rather than the standard Z-score map was small, and the depicted range was different.

The Z-score map analyzed the E.CAM AD data in combination with the E.CAM NDB. Then, SVA analyzed, calculated Se, Ex and Ratio are standards of this examination. Regarding the scores of the “Se” and “Ex”, the most approximate compensation to the standard of the present study (Fig. 5c; Se = 2.05, Ex = 47.08) was the compensation using Normal-SPECT data (Se = 1.95, Ex = 44.01). This was followed in the order of the compensation using 3D-Brain data (Se = 1.93, Ex = 43.50), the compensation using Hoffman data (Se = 1.79, Ex = 37.32), the compensation using pool data (Se = 1.60, Ex = 29.07), and no compensation (Se = 1.38, Ex = 20.68). Regarding the scores of “Ratio”, the most approximate compensation to the standard in the present study (Fig. 5c, 15.16) was the compensation using Hoffman data (17.30). When we did not revise the difference in the cameras, the ratio was 18.45. When we used Normal-SPECT data (31.06) for compensation for the difference in the cameras, the ratio showed a larger than normal value. In contrast, the “Ratio” of compensation using 3D-Brain data (7.23) and that of compensation using pool data (8.99) was lower than that of the standard in the present study. Also, “Ratio” acquired by the analysis using IRIX NDB on the IRIX AD data was 2.8 times higher than that of the standard in the present study.

### Examination when healthy subject data group is different from a group constituting Normal-SPECT data

**Fig. 6:**
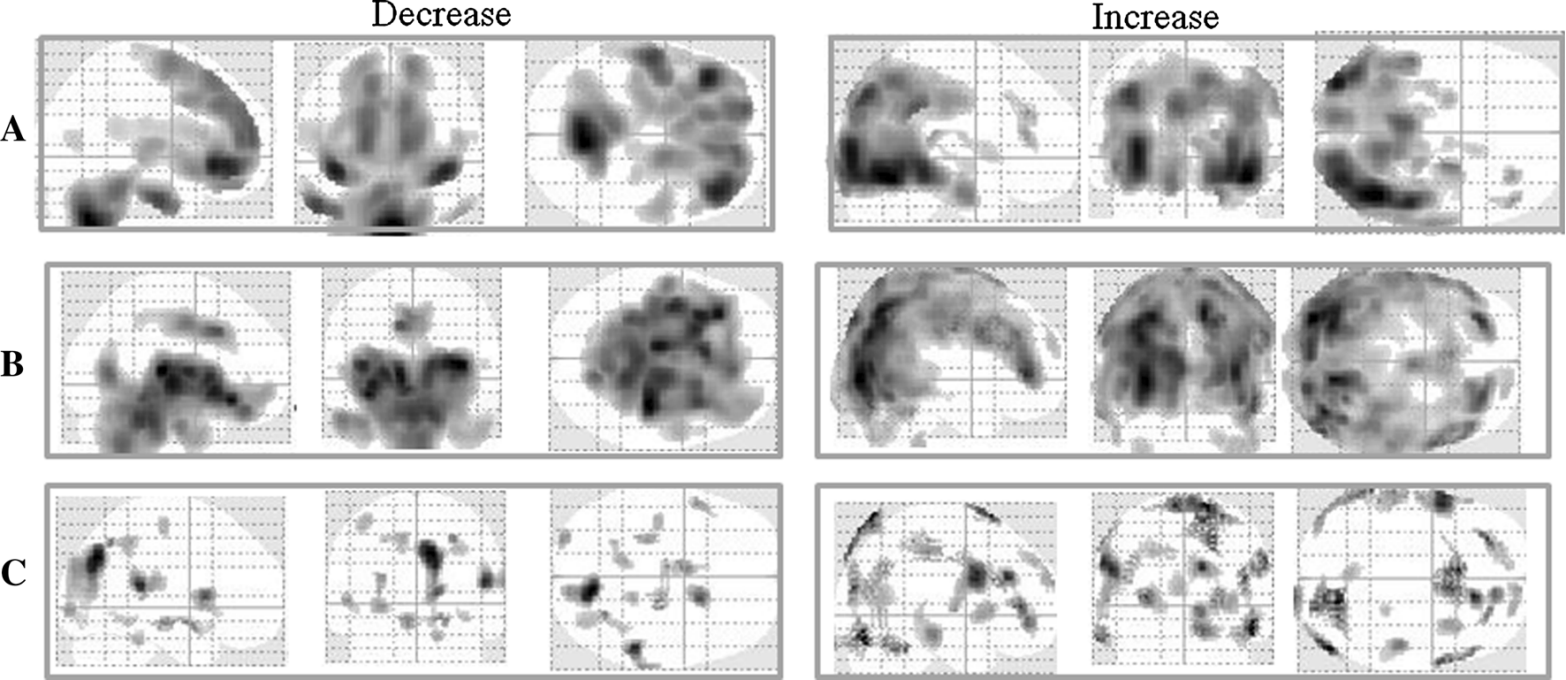
Maximum intensity projection (MIP) of SPM8 result for each two-sample *t* test on basis of E.CAM Ref PAO data by ^99m^Tc-HMPAO SPECT. MIPs show the increase and decrease for IRIX data (**a**), IRIX AC^−^ data (**b**), and compensated IRIX AC^−^ data by Normal-SPECT (**c**), respectively

Figure 6a, shows the results of the two-sample *t* test between the E.CAM Ref data of ^99m^Tc-HMPAO (referred to below as E.CAM Ref PAO data) and the IRIX data of ^99m^Tc-HMPAO. “Decrease” means the region in which the counts of IRIX data are lower than those of the E.CAM Ref PAO data. And “Increase” means the region in which the counts of the IRIX data are higher than those of the E.CAM Ref PAO data. The counts of the IRIX data were lower than those of the E.CAM Ref PAO data in the frontal lobe and higher than those of the E.CAM Ref PAO data in the parietal lobe, temporal lobe and occipital lobe.

The counts of the IRIX AC^−^ data were significantly lower than those of the E.CAM Ref PAO data in the central area and were significantly higher than those of the E.CAM Ref data in the outside area (Fig. 6b). Figure 6c, shows the results of the two-sample *t* test between IRIX AC^−^ data that were compensated for using Normal-SPECT data and E.CAM Ref PAO data. The difference in count of the “Decrease” in the central area and the difference in count of the “Increase” in the outside area decreased. Therefore, the difference-compensation effect of the cameras is good.

## Discussion

There was a statistically significant difference between the SPECT counts of the same subject depending on the cameras. The statistical significance was not scattered, but occupied a specific region such as the frontal lobe (Fig. 2a). Therefore, some compensation for the difference in cameras is necessary to share an NDB in the eZIS analysis. If a result of the eZIS analysis varies according to the difference of the device and cannot be diagnosed definitely, this is because it is a problem.

In the present study, IRIX AC^−^ data approximated the E.CAM Ref ECD data because of compensation. However, according to the examinations using the Hoffman by Yamamoto [22] and Haramoto [23], a satisfactory effect of the compensation was not achieved in all brain regions. In contrast, a satisfactory effect was achieved in the compensation by Normal-SPECT data because the difference in count decreased in “Decrease image” and “Increase image”. A stable compensation effect was provided in Central area and outside area. By the compensation using Phantom data including Hoffman, there is an area in which a good compensation effect is not achieved.

The above result was also proven by the assessment of the effect of the compensation by Z-score (Fig. 3; Table 1). A satisfactory effect of the compensation could be achieved only in compensation using Normal-SPECT data in both the central and marginal areas. In the compensations using the other phantom data, the central area was inconsistent with the marginal area and “Decrease” was inconsistent with “Increase”. This is due to the difference in the formulations distribution, which arose from the difference in structure between the phantom data and actual brain tissue because the image reconstruction conditions were set so that the resolutions were the same in each SPECT image of the two cameras [19]. The Z-score acquired by eZIS analysis using the E.CAM NDB on the IRIX data without compensation, IRIX data (No cor), was more approximate to the standard Z-score than the compensation using Phantom data. Therefore, although the cameras differed, if we equalize the image reconstruction conditions of the collection camera (the camera used for the clinical study) and those of the standard camera (the camera that created the NDB), we were able to acquire data that better approximated the E.CAM Ref ECD data than the data acquired by the compensation for the difference in cameras using phantom data.

Figure 4 and Table 2, show the results of the assessment of the correction effect of compensation by the correlation coefficient of SPECT counts, with the correlation coefficient between the E.CAM Ref ECD data and IRIX data (No cor) higher than the correlation coefficient between the E.CAM Ref ECD data and IRIX data compensated by phantom data. Suggested by the examination using Z-score as mentioned above, equalizing the image reconstruction conditions of the collection camera (the camera used for the clinical study) and those of the standard camera (camera that created the NDB) is more effective than the compensation of the difference in the cameras using phantom data. We do not compensation for the difference in the cameras, but use the NDB.

The effect of the compensation using Normal-SPECT data was satisfactory because the correlation coefficients were the highest in all regions. In contrast, the correlation coefficients of the compensation using pool data were the lowest in all regions except for the sub-lobar. Among these regions, in the anterior lobe, the correlation coefficient was especially low. The anterior lobe was the region in which the difference in count was large in the compensation by pool data (Fig. 2e). This meant that the effect of the compensation by pool data was restrictive.

The eZIS analyzed E.CAM AD data in combination with the E.CAM NDB. Then, we performed SVA analysis. And calculated “Se and Ex” are standards. Se and Ex of the compensation using 3D-Brain data (Fig. 5f) were the most approximate to the standard among those of the compensations using the other Phantom data. However, the ratio was the smallest among all compensations. This meant the existence of many voxels with Z-score ≥2 produced by the compensation in the Z-score map. Therefore, the 3D-Brain data were not appropriate data for the compensation. Similarly, in the compensations using pool data, Ratios were small; therefore, pool data were not appropriate data for compensation. In the compensation using Hoffman data (Fig. 5e), Se and Ex were approximate to the standard scores. This result was more satisfactory than the case of without compensation (Fig. 5d). For the reasons stated above and the result of SVA analysis, among three Phantom data, only Hoffman data were appropriate for the compensation. We are satisfied with the result of the SVA analysis, but are not satisfied with the result of the eZIS analysis. If we use it for compensation for the difference in the cameras by Phantom data, we think that it is Hoffman data.

3D-Brain is the phantom which best simulated the brain of humans, but only the gray matter area that can contain a radionuclide. On the other hand, Hoffman employs a gap between the plates of the 89 slices but allows for a white matter area and a gray matter area. We infer that it is nearer to the distribution of the human brain. In other words, distribution rather than perfect structure is more necessary for compensation.

In Fig. 6a, we show the differences in the SPECT image due to the camera in the case of ^99m^Tc-HMPAO. The “Decrease” image and “Increase” image in the case of ^99m^Tc-HMPAO showed the same tendency as in the case of ^99m^Tc-ECD (Fig. 2a). It has been reported that both ^99m^Tc-HMPAO distributions and ^99m^Tc-ECD distributions in the brain are the same a few hours after administration [24–28]. Therefore, this statistical significance clearly shows the differences in the SPECT image by the camera. When we use Normal-SPECT data for compensation for the difference in the cameras: When radiopharmaceutical compares the case of ^99m^Tc-HMPAO (Fig. 6c) and the case of ^99m^Tc-ECD (Fig. 2f), in the case of ^99m^Tc-HMPAO, the regions with significant differences were mostly scattered throughout the brain. On the other hand, the statistical significance of the central area (Fig. 6b) disappeared; therefore we were able to obtain a satisfactory compensation effect. However, for the compensation using Normal-SPECT data, we must collect the same normal subject’s raw data by both the camera that created the NDB and the camera used for the clinical study. This procedure is more difficult than constructing an NDB.

Therefore, in future work, we will study ways to conveniently construct the data for the compensation of the difference in the collection camera by extracting a control group from the clinical data obtained with each camera. This control group should consist of over 25 cases, which would be regarded as a large sample.

## Conclusions

We verified differences in SPECT images of cerebral blood flow caused by a difference in the imaging camera. This has been already checked, but we inspected it again. Differences were noted in the frontal lobe, parietal lobe, temporal lobe, and occipital lobe when we compared the SPECT image of E.CAM with the SPECT image of the IRIX. Compensation for the difference in the cameras using pool data in the eZIS analysis was not able to achieve a good compensation effect in all of the end-points examined in this study. Excellent compensation was achieved using Hoffman data rather than 3D-Brain data. The effect of the compensation for the difference in the cameras using Normal-SPECT data was superior to the compensation effect using each of the three Phantom data sets.
